# A geometric approach to quantifying the neuromodulatory effects of persistent inward currents on individual motor unit discharge patterns

**DOI:** 10.1088/1741-2552/acb1d7

**Published:** 2023-01-30

**Authors:** James A Beauchamp, Gregory E P Pearcey, Obaid U Khurram, Matthieu Chardon, Y Curtis Wang, Randall K Powers, Julius P A Dewald, CJ Heckman

**Affiliations:** 1 Department of Biomedical Engineering, McCormick School of Engineering, Northwestern University, Chicago, IL, United States of America; 2 Department of Physical Therapy and Human Movement Sciences, Feinberg School of Medicine, Northwestern University, Chicago, IL, United States of America; 3 School of Human Kinetics and Recreation, Memorial University of Newfoundland, St. John’s, NL, Canada; 4 Department of Physiology and Biomedical Engineering, Mayo Clinic, Rochester, MN, United States of America; 5 Northwestern Argonne Institute for Science and Engineering (NAISE), Northwestern University, Evanston, IL, United States of America; 6 Department of Neuroscience, Feinberg School of Medicine, Northwestern University, Chicago, IL, United States of America; 7 Department of Electrical and Computer Engineering, California State University, Los Angeles, Los Angeles, CA, United States of America; 8 Department of Physiology and Biophysics, University of Washington School of Medicine, Seattle, WA, United States of America; 9 Department of Physical Medicine and Rehabilitation, Feinberg School of Medicine, Northwestern University, Chicago, IL, United States of America; 10 Shirley Ryan AbilityLab, Chicago, IL, United States of America

**Keywords:** persistent inward currents, motor units, monoamines

## Abstract

*Objective.* All motor commands flow through motoneurons, which entrain control of their innervated muscle fibers, forming a motor unit (MU). Owing to the high fidelity of action potentials within MUs, their discharge profiles detail the organization of ionotropic excitatory/inhibitory as well as metabotropic neuromodulatory commands to motoneurons. Neuromodulatory inputs (e.g. norepinephrine, serotonin) enhance motoneuron excitability and facilitate persistent inward currents (PICs). PICs introduce quantifiable properties in MU discharge profiles by augmenting depolarizing currents upon activation (i.e. PIC amplification) and facilitating discharge at lower levels of excitatory input than required for recruitment (i.e. PIC prolongation). *Approach*. Here, we introduce a novel geometric approach to estimate neuromodulatory and inhibitory contributions to MU discharge by exploiting discharge non-linearities introduced by PIC amplification during time-varying linear tasks. In specific, we quantify the deviation from linear discharge (‘brace height’) and the rate of change in discharge (i.e. acceleration slope, attenuation slope, angle). We further characterize these metrics on a simulated motoneuron pool with known excitatory, inhibitory, and neuromodulatory inputs and on human MUs (number of MUs; Tibialis Anterior: 1448, Medial Gastrocnemius: 2100, Soleus: 1062, First Dorsal Interosseus: 2296). *Main results*. In the simulated motor pool, we found brace height and attenuation slope to consistently indicate changes in neuromodulation and the pattern of inhibition (excitation–inhibition coupling), respectively, whereas the paired MU analysis (Δ*F*) was dependent on both neuromodulation and inhibition pattern. Furthermore, we provide estimates of these metrics in human MUs and show comparable variability in Δ*F* and brace height measures for MUs matched across multiple trials. *Significance*. Spanning both datasets, we found brace height quantification to provide an intuitive method for achieving graded estimates of neuromodulatory and inhibitory drive to individual MUs. This complements common techniques and provides an avenue for decoupling changes in the level of neuromodulatory and pattern of inhibitory motor commands.

## 1. Introduction

Motor units (MUs), each comprising a single spinal motoneuron and the set of muscle fibers it innervates, transform motor commands into muscle twitches and serve as the final common pathway (Sherrington 1907) of the nervous system. The process within a MU from descending excitatory command to muscle action potential is thought to represent a non-linear transfer function, where ionotropic excitatory and inhibitory synaptic inputs are modified by the action of metabotropic neuromodulatory inputs (Lee and Heckman 2000, Powers and Binder 2001, Heckman and Enoka 2012, Binder *et al* 2020). These neuromodulatory inputs, consisting of brainstem-derived neurotransmitters (e.g. norepinephrine, serotonin), enhance motoneuron excitability by acting on G-protein coupled receptors to alter membrane potentials, firing thresholds, and facilitate persistent inward Na and Ca currents (PICs) (Elliott and Wallis 1992, Hsiao *et al* 1997, Wada *et al* 1997, Lee and Heckman 1999a, 2000, Perrier and Hounsgaard 2003, Fedirchuk and Dai 2004, Harvey *et al* 2006a). PICs are excitatory dendritic currents mediated by voltage-sensitive channels that provide motoneurons with an intrinsically driven source of current. This additional current introduces observable non-linearities in motoneuron discharge by augmenting excitatory depolarizing currents upon activation (i.e. PIC amplification) and facilitating discharge at lower levels of excitatory input than required for a motoneurons recruitment (i.e. PIC prolongation) (Hounsgaard and Kiehn 1985, Crone *et al* 1988, Hounsgaard *et al* 1988, Lee and Heckman 1998a, Heckman *et al* 2008). With these actions manifest in the discharge pattern of MUs and facilitated by modulatory neurotransmitters, the discharge nonlinearities introduced by PICs offer a window into the commands underlying human motor control (Johnson *et al* 2017, Khurram *et al* 2022). Quantifying the actions of PICs may grant insight into how neuromodulatory inputs sculpt motor commands for voluntary movement and could confer logic to motor impairments observed in pathological conditions where neuromodulatory inputs are theorized dysfunctional (e.g. spinal cord injury, chronic stroke) (Murray *et al* 2010, Mcpherson *et al* 2018, Li *et al* 2019, Beauchamp *et al* 2022).

Though direct quantification of motor commands in humans has proven elusive, where intracellular recordings remain impossible, profiles of MU discharge rate retain the hallmark amplification and prolongation indicative of PICs. In humans, amplification, or PIC acceleration of discharge rate, is observed as a nonlinear relationship between a MU’s discharge profile and the resultant force or joint torque during isometric contractions of linearly increasing intensity (Person and Kudina 1972, De Luca *et al* 1982, Kiehn and Eken 1997, Gorassini *et al* 1998, 2002, Walton *et al* 2002, Udina *et al* 2010, Fuglevand *et al* 2015, Khurram *et al* 2022). This nonlinearity parallels observations in animal preparations where MUs display an initial steep increase in discharge rate upon activation, indicating intrinsic activation of the motoneurons from PICs, followed by a period of attenuated increase. Prolongation of discharge from PICs, on the other hand, typically manifests as a discrepancy between the synaptic input required for recruitment and derecruitment of MU discharge during linear isometric ramp contractions. This recruitment and de-recruitment mismatch, or hysteresis, is commonly attributed to persistent depolarizing currents supplied by PICs. Together, the amplification and prolongation of MU discharge induced by PICs yield quantifiable properties in the discharge profile of a MU that can be used to estimate neuromodulatory motor commands.

A well-established approach for estimating the magnitude of PICs in MUs includes a paired MU analysis, which quantifies Δ*F*, and represents the discharge rate hysteresis (i.e. PIC prolongation) of a higher threshold MU (test unit) with respect to a lower threshold MU (reporter unit). In this paradigm, the reporter MU serves as a proxy for synaptic drive and is used to estimate the difference in excitatory input required at recruitment and de-recruitment of the test MU (Gorassini *et al* 1998, 2002, Powers and Heckman 2015). This method is commonly employed, has been extensively validated, and is insightful under most circumstances, but Δ*F* by its definition is a paired MU analysis technique that is influenced by the properties of two MUs. Thus, values of Δ*F* may be confounded across recruitment threshold and any observed changes following interventions or varying conditions cannot be isolated to alterations in either the discharge rate profile of the reporter MU or hysteresis of the test unit.

Less effort has been placed on quantifying the non-linearity in discharge rate following MU recruitment during linear time-varying tasks (i.e. PIC amplification). Several groups have made notable efforts to quantify this non-linearity by fitting ascending phases of MU discharge with either an exponential or linear function and dichotomizing MUs based on fit error (De Luca and Contessa 2012, Fuglevand *et al* 2015, Revill and Fuglevand 2017). Practitioners of this method conclude that MUs exhibiting a lower fit error with an exponential function behave less linearly and are likely under greater influence from PICs. Although insightful, dichotomizing the data unnecessarily removes the ability to detect graded changes in PICs from alterations in neuromodulatory or inhibitory inputs. A graded approach capable of isolating estimates to single MUs would help provide detailed insight into the neuromodulatory control of motor output.

Current methods used to quantify the effects of PICs on MUs have facilitated insight into both healthy and pathological motor control but leave potential for improvement. To provide a measure of neuromodulatory drive to MUs on a single unit level, we propose a geometric approach for quantifying non-linearity in MU discharge rates during linear increases in voluntary effort. In the absence of PICs, MUs behave as passive integrators of synaptic drive, and thus deviations from linearity during linear time-varying tasks should represent suprathreshold depolarizing currents from PICs. Therefore, the maximum magnitude of deviation in discharge rate (referred to here as brace height) from a theoretical linear increase in discharge rate, from recruitment to peak, can be used as a proxy for PIC amplification. Furthermore, given that this maximum deviation is geometrically the point at which the change in discharge rate over time transitions from a trend of steep increase to an attenuated increase, this point can be used to separate and characterize the secondary and tertiary discharge ranges (Afsharipour *et al* 2020, Mcauliffe *et al* 2020). The work herein defines the quantification of brace height and these associated metrics; validates and compares these metrics on a simulated motoneuron pool with known excitatory, inhibitory, and neuromodulatory inputs; characterizes these metrics on a large dataset of human MUs; and discusses potential implications and limitations of the approach.

## 2. Methods

### 2.1. Simulated motor pool

#### 2.1.1. Model motoneurons

Simulated motoneuron spike trains were obtained from a pool of motoneurons modeled with methods similar to those discussed previously (Powers and Heckman 2017). This motoneuron pool consists of 20 model motoneurons to reflect the typical sample size of MUs discriminated with high-density surface electromyography (EMG) recordings, with each motoneuron possessing a range of intrinsic properties. Each model motoneuron consisted of a soma compartment and four dendritic compartments, each coupled to the soma. The size of the compartments, the values of capacitance and passive conductance of each compartment, and the values of coupling conductance between compartments were chosen to replicate several features of fully constructed dendritic trees, including local input resistance, asymmetric voltage attenuation between the soma and dendritic areas, membrane time constants, total surface area, and neuronal input resistance, as described by Kim and colleagues (Kim *et al* 2009). Spike conductances (Na and K) and conductances mediating the medium afterhyperpolarization (AHP) were inserted into the soma compartment and a Ca conductance mediating the slowly-activating PIC was inserted into each of the dendritic compartments. In addition, a hyperpolarization-activated mixed-cation (HCN) conductance was inserted into all compartments. Conductance densities, kinetics, and steady-state activation curves were originally tuned to recreate the range of input–output behavior recorded in medial gastrocnemius (MG) motoneurons in decerebrate cats, as described previously (Powers and Heckman 2017). Specifically, the models replicated the range of current–voltage (*I*–*V*) relations recorded during somatic voltage-clamp and the frequency–current (*F*–*I*) relations recorded during somatic current injection. The range of observed behaviors was achieved by systematically varying several parameters across the pool, including AHP duration, specific input resistance, surface area, and the density and half-activation voltage of the PIC conductance.

Since we intend to compare the results of these simulations to the discharge behavior of human MUs, we have implemented modifications to the parameters of the model to better represent the behavior observed with human subjects. Explicitly, the discharge behavior of human MUs during moderate voluntary contractions, when compared to cat MG motoneurons that the original model is based upon, generally exhibit lower firing rates and only partial recruitment of the motor pool. To better represent these differences in the model, we made four modifications to the parameters. First, we restricted the range of values governing excitability to the first 75% of the original recruitment range (i.e. the parameters of motoneuron 20 in the new model corresponded to motoneuron 15 in the original parameter set). Second, AHP durations and amplitudes were increased by increasing the values of the time constant of calcium removal for the calcium-activated potassium conductance (from 60–10 ms in the original model to 90–57 ms). The larger and longer AHPs acted to oppose early PIC activation during increasing excitatory synaptic drive, so we hyperpolarized the PIC half-activation threshold (from −40 to −37 mV in the original model to −42 to −40.4 mV). Finally, in the original model, the slow decay of PICs observed in high threshold MG motoneurons (Lee and Heckman 1996) was replicated by including a voltage-dependent inactivation process. We found that this process limited the firing rate hysteresis to values below those typically seen in human MU recordings, so we eliminated PIC inactivation in the present model pool.

#### 2.1.2. Neuromodulatory and inhibitory inputs

To compare changes in the proposed metrics as a function of the composition of motor commands to the pool, spike trains were generated at various levels of neuromodulatory drive and patterns of inhibition (excitation–inhibition coupling). Neuromodulatory inputs were adjusted by multiplying the max conductance of the L-type calcium channel by a multiplier (0.8, 0.9, 1.0, 1.1, 1.2) to either increase or decrease the relative magnitude of PICs. The pattern of inhibition was adjusted as a function of excitation through dendritic inhibitory conductance. Specifically, inhibitory conductance was adjusted to recreate an inhibitory input that positively scaled with the excitatory input (proportional) or an inhibitory input that negatively scaled with excitatory input (reciprocal). For all simulations, models were driven with noisy (Gaussian) excitatory conductance commands that increased and decreased, reaching peak conductance in 10 s, as described previously (Powers and Heckman 2015, 2017). Values of the inhibitory profile are reported from −0.7 to 0.7 and correspond to reciprocal and proportional, respectively, with the value indicating the proportion of inhibition to excitation (i.e. 0.7: peak inhibition conductance ∼70% of excitation). An inhibitory profile of 0.0 indicated a constant inhibitory offset. This constant offset was present in all inhibitory profile cases and chosen as a constant inhibitory conductance that ensured all model neurons ceased discharge following the cessation of excitatory input in the 1.0 neuromodulation state.

All simulations were run using NEURON software (Hines and Carnevale 1997). NEURON files specifying motoneuron pool parameters, conductance mechanisms, and protocols for producing motoneuron pool output in response to synaptic conductance inputs can be found at http://modeldb.yale.edu/239582.

### 2.2. Human MUs

#### 2.2.1. Participants

MU spike trains were obtained from two participant cohorts, pertaining to distal muscles of either the lower or upper limb. This included 30 participants in total; 21 participants (F: 5, M: 16; age: 26.4 ± 1.7) in the lower limb cohort and nine participants (F: 2, M: 7; age: 27.9 ± 5.5) in the upper limb cohort. Participants reported no known neuromuscular, musculoskeletal, or cardiovascular impairments and provided written and informed consent in accordance with the Declaration of Helsinki. Portions of the data collected in the lower limb cohort have been previously reported elsewhere and are used here as a secondary analysis (Beauchamp *et al* 2022, Pearcey *et al* 2022).

#### 2.2.2. Overview

For both upper and lower limb cohorts, we asked participants to generate isometric joint torque contractions in the shape of a linear triangular ramp to 30% of their maximum ability. This paradigm consists of a linear increase and subsequent decrease in joint torque and is commonly used in the field (De Luca *et al* 1982, Farina *et al* 2009, Oya *et al* 2009, Kim *et al* 2020, Hassan *et al* 2021, Orssatto *et al* 2021). In the lower limb, participants generated ramp contractions through either ankle dorsiflexion or plantarflexion with grid electrodes placed atop the skin overlying the tibialis anterior (TA), MG, and soleus (SOL) muscle bellies. In the upper limb, participants generated ramp contractions through index finger abduction with a grid electrode overlying the muscle belly of the first dorsal interosseus (FDI).

#### 2.2.3. Experimental setup

For all experimental sessions, participants were first seated in a Biodex chair, with shoulder and waist restraints used to prevent movement. In the lower limb cohort, a participant’s left foot was then securely attached to a footplate fixed onto a Systems 4 Dynamometer (Biodex Medical Systems, Shirley, NY), with their ankle joint coincident to the axis of rotation of the dynamometer, their hips at approximately 80° of flexion, their left knee at 20° flexion, and their left ankle at 10° of plantarflexion. In the upper limb cohort, a participant’s left forearm was cast and fixed to a rigid horizontal aluminum plate while their left index finger was held parallel to their forearm and placed perpendicular to a linear load cell (LCFD-25, Omega Engineering, Norwalk, CT) at the proximal interphalangeal joint. Throughout the experimental session, we maintained a participant’s upper limb in 90° of elbow flexion, 40° of shoulder flexion, and 80° of shoulder abduction. Target effort ramps (i.e. dorsiflexion/plantarflexion torque or index finger abduction force) were provided on a television screen via a custom interface (MATLAB (R2020b), The Mathworks Inc., Natick, MA), with collected torque/force smoothed by a 125 ms moving average window before being provided as visual feedback to the participant. For subsequent analysis, raw signals were digitized (2048 Hz) and lowpass filtered (50 Hz) with a fifth-order Butterworth filter.

#### 2.2.4. Experimental protocol

At the onset of each experimental session, we asked participants to first generate maximal voluntary isometric contractions in the direction of interest for that given session. This included either maximum dorsiflexion and plantarflexion or maximum index finger abduction. A minimum of two maximal contractions were performed, with at least 1 min of rest separating contractions, and repeated until the peak torque/force within the last contraction deviated by less than 10% of the previous contraction. We then used the maximum voluntary torque/force (MVT/F) achieved during these contractions to normalize all subsequent ramp contractions. Each ramp contraction started from rest and consisted of a 10 s linear increase to 30% MVT and a 10 s decrease back to rest (i.e. 3% MVT/s rise and decay speeds). To mitigate potential learning effects and ensure smooth contractions, participants completed a minimum of six practice ramps. Following practice trials, each experimental session consisted of 4–12 ramp contractions for each target direction.

#### 2.2.5. MU decomposition

In the lower limb, high-density surface EMG (HD-sEMG) was collected via 64 channel electrode grids (GR08MM1305, OT Bioelettronica, Turin, IT) placed atop the skin overlying the TA, MG, and SOL muscle bellies with adhesive foam and conductive paste. In the upper limb cohort, HD-sEMG was similarly collected via 64 channel electrode grids (GR04MM1305, OT Bioelettronica, Turin, IT) placed atop the skin overlying the FDI. Before electrode placement, the muscles of interest were identified by experienced investigators and the skin overlying the muscle was shaved, abraded with abrasive paste, and cleaned with isopropyl alcohol. In the lower limb cohort, two Ag/AgCl ground electrodes were placed bilaterally on the right and left patella and a moist band electrode was placed around the right ankle. In the upper limb cohort, one Ag/AgCl electrode was placed on the left acromion. All HD-sEMG signals were acquired with differential amplification (150×), bandpass filtered (10–900 Hz), and digitized (2048 Hz) using a 16-bit analog-to-digital converter (Quattrocento, OT Bioelettronica, Turin, IT).

Following collection, each channel of surface EMG was bandpass filtered at 20–500 Hz (second‐order, Butterworth) and visually inspected to remove channels with substantial artifacts, noise, or saturation of the A/D board. The remaining EMG channels were decomposed into individual MU spike trains using convolutive blind source separation and successive sparse deflation improvements (Negro *et al* 2016, Martinez-Valdes *et al* 2017). The silhouette threshold for decomposition was set to 0.87. To improve decomposition accuracy and correct spikes that indicated non-physiological MU discharge, experienced investigators conducted manual editing of the spike trains. Specifically, automatic decomposition results were improved by iteratively re-estimating the spike train and correcting for missed spikes or substantial deviations in the discharge profile (Boccia *et al* 2019, Del Vecchio *et al* 2020, Hug *et al* 2021).

### 2.3. Metrics

#### 2.3.1. Pre-processing

Before quantification of the following metrics, discrete estimates of instantaneous discharge rate were generated from decomposed binary MU spike trains and smoothed with support vector regression (SVR) to create a continuous estimate of discharge rate for each MU, as previously described (Beauchamp *et al* 2022). In brief, estimated MU discharge times were obtained from decomposed MU spike trains and used to quantify the inter-spike interval (ISI), or the time between each consecutive spike. Discrete estimates of instantaneous discharge rate were then calculated as the reciprocal of the time series ISI for each MU and used to train an SVR model. Specifically, we used Matlab’s inbuilt function *fitrsvm* to train an SVR model with L1 soft-margin minimization to predict instantaneous discharge rate as a function of the corresponding time instances for each MU (MATLAB (R2020b), The Mathworks Inc., Natick, MA). Smooth estimates of discharge rate were then generated with Matlab’s inbuilt *predict* function, along a time vector from MU recruitment to derecruitment sampled at 2048 Hz (MATLAB (R2020b), The Mathworks Inc., Natick, MA). Hyperparameters were chosen in accordance with those previously suggested (Beauchamp *et al* 2022). For both upper and lower limb cohorts, MUs with less than ten consecutive discharge instances were excluded from further analysis. This yielded 1448 MUs in the TA, 2100 in the MG, 1062 in the SOL, and 2296 in the FDI.

#### 2.3.2. Brace height

**Figure 1.**
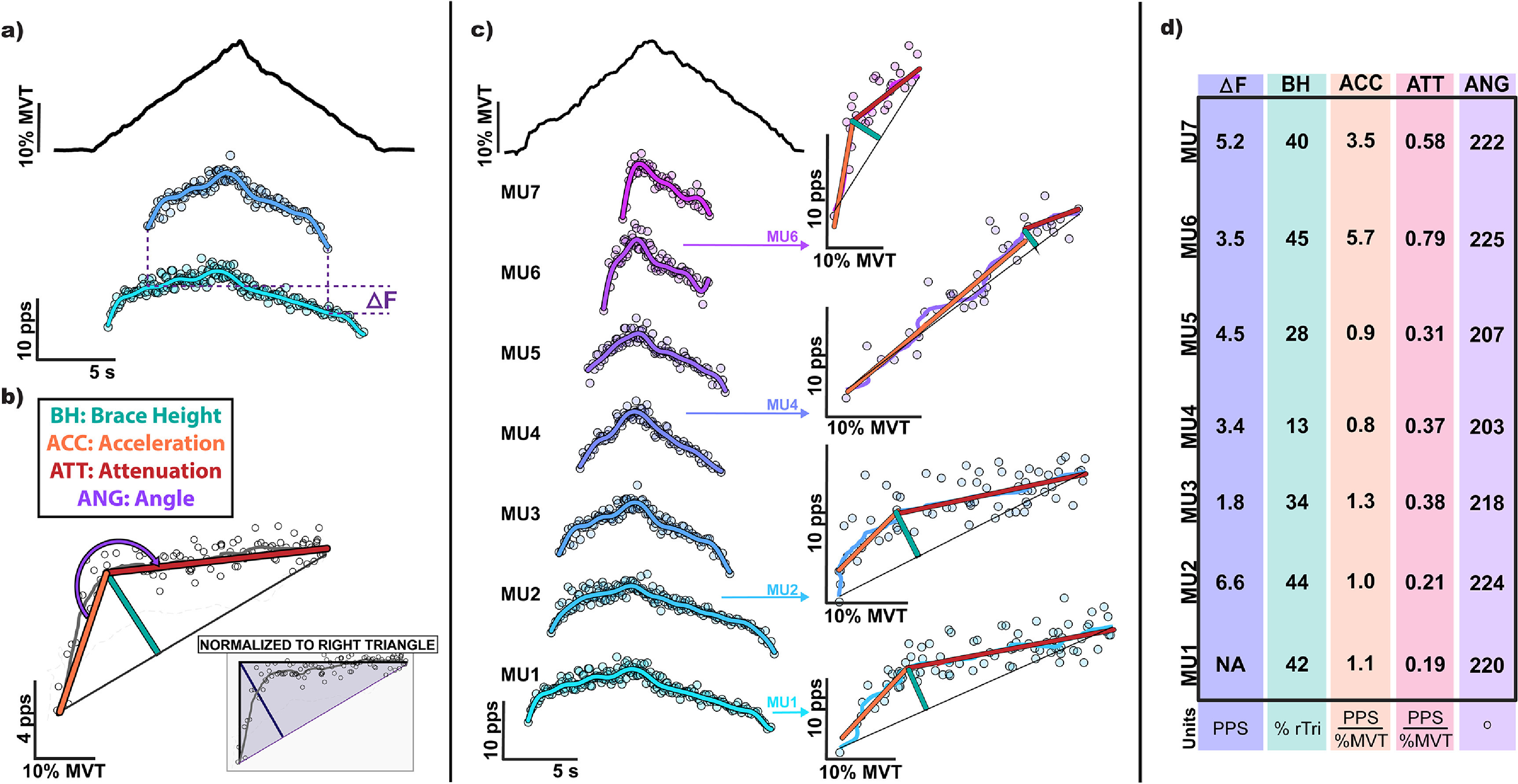
The employed methods for quantifying neuromodulatory effects on motor units (MUs), including the (a) paired motor unit analysis technique (Δ*F*), and the proposed (b) brace height and its associated metrics. Of note, we have normalized brace height values to the height of a right triangle whose hypotenuse represents a linear line from recruitment to peak discharge. This right triangle represents the brace height that would be seen under a theoretical situation where full PIC activation achieves MU excitation sufficient to reach peak discharge and saturate the MU. This makes brace height in units of the percentage of that achieved with a right triangle (% rTri). All metrics are further shown (c–d), quantified for a random sampling of MUs decomposed from the medial gastrocnemius during a linear plantarflexion ramp contraction to 30% of an individual’s maximum voluntary torque (MVT). A summary of each metric for the sampled MUs is shown in d. (pps: pulse per second; s: second).

To provide an estimate of PICs and neuromodulatory drive to MUs on a single unit level, we used a pseudo-geometric approach to quantify the amplification of discharge rate following the onset of MU recruitment. This non-linearity in discharge following MU onset is a product of intrinsic activation from PICs and should therefore correlate with neuromodulatory drive, given the known dependence of PICs on monoamines (Wada *et al* 1997, Lee and Heckman 1999a, Perrier and Hounsgaard 2003, Harvey *et al* 2006a). For each MU, we take the smoothed trace of MU discharge and plot this as a function of the joint torque/force produced during the contraction. A theoretical linear discharge trace is then generated, fitting a straight line from the discharge rate at MU recruitment to peak discharge rate. Brace height is then quantified as the magnitude of the maximum orthogonal vector from this straight line to the smooth MU discharge trace, as shown in figure 1. To account for the scaling of brace height with discharge range (i.e. rate modulation, difference in discharge rate from recruitment to peak) and ensure brace height represents the relative deviation from linearity, we have chosen to normalize brace height to the height of a right triangle whose hypotenuse represents a straight line from recruitment to peak discharge (figure 1(b)). This value represents the brace height that would be seen under a theoretical situation where full PIC activation achieves MU excitation sufficient to reach peak discharge and saturate the MU.

In addition to providing insight into PIC amplitude, the process of brace height quantification yields supplemental metrics that could facilitate a deeper analysis of individual MU discharge profiles. Given the inherent definition of brace height, its instance of occurrence geometrically corresponds to the point at which the change in discharge rate over time transitions from a trend of steep increase to an attenuated increase. Therefore, this point can be used to segment the ascending phase of MU discharge into two regions of interest: an acceleration phase and an attenuation phase (figure 1(b)), which correspond to the secondary and tertiary discharge ranges, respectively. The initial acceleration phase (i.e. secondary range) corresponds to a region of discharge where the gain from MU input to output is high, presumably from amplification by PICs. Conversely, during the attenuation phase, the gain from MU input to output is decreasing and further increases in effort produce attenuated increases in discharge rate. This phase corresponds to a region of MU discharge where PICs are likely fully activated, and the MU is approaching peak discharge. Though many metrics could be drawn from these regions, we have chosen to quantify the slope for both phases as well as the angle between these two phases to compare against brace height and Δ*F*. For all metrics, MUs that exhibited a negative acceleration slope, had a normalized brace height greater than 200%, or had an occurrence of peak discharge after peak torque, were investigated for irregularities in torque or discharge profile and removed from further analysis.

#### 2.3.3. Paired MU analysis

Δ*F* is a commonly employed metric used to estimate the magnitude of PICs and represents the hysteresis of a higher threshold MU with respect to the discharge rate of a lower threshold unit. Δ*F* for a given MU (test unit) is quantified as the change in discharge rate of a lower threshold MU (reporter unit) between the recruitment and derecruitment instance of the test unit. To account for the possible pairing of a test unit with multiple lower threshold reporter units, we represented Δ*F* for a given test unit as the average change in discharge rate across all reporter unit pairs. To ensure that MU pairs likely received a common synaptic drive, we only included test unit-reporter unit pairs with rate-rate correlations of *r* ^2^ > 0.7 (Gorassini *et al* 2002, Udina *et al* 2010, Wilson *et al* 2015). To allow for full activation of the PIC in the reporter unit, we excluded any pairs with recruitment time differences <1 s (Bennett *et al* 2001, Powers *et al* 2008, Hassan *et al* 2020). Furthermore, to avoid saturated reporter units, we excluded test unit-reporter unit pairs in which the reporter unit discharge range was <0.5 pps while the test unit was active (Stephenson and Maluf 2011).

### 2.4. Matched MU analysis

To compare the stability of the proposed metrics over time on repeated observations of the same MU, we matched decomposed MUs from the human TA trials to identify repeated observations of the same MU across trials. To do this, we estimated MU action potential waveforms (MUAPs) with spike triggered averaging and computed a 2D cross correlation between the spatial representation of the MUAPs between ramp contraction trials (Martinez-Valdes *et al* 2017, Del Vecchio *et al* 2019). For each MU, this was repeated across all MUs in successive contractions. We then inspected the normalized correlation values between MU pairs and deemed those greater than 0.8 a matched unit, with matched unit pairs across trials collapsed and given a single unique MU identifier. We then collected the values for each of the proposed neuromodulatory metrics for these matched MUs and used them to investigate and compare each metrics repeatability.

### 2.5. Statistical approach

**Figure 2.**
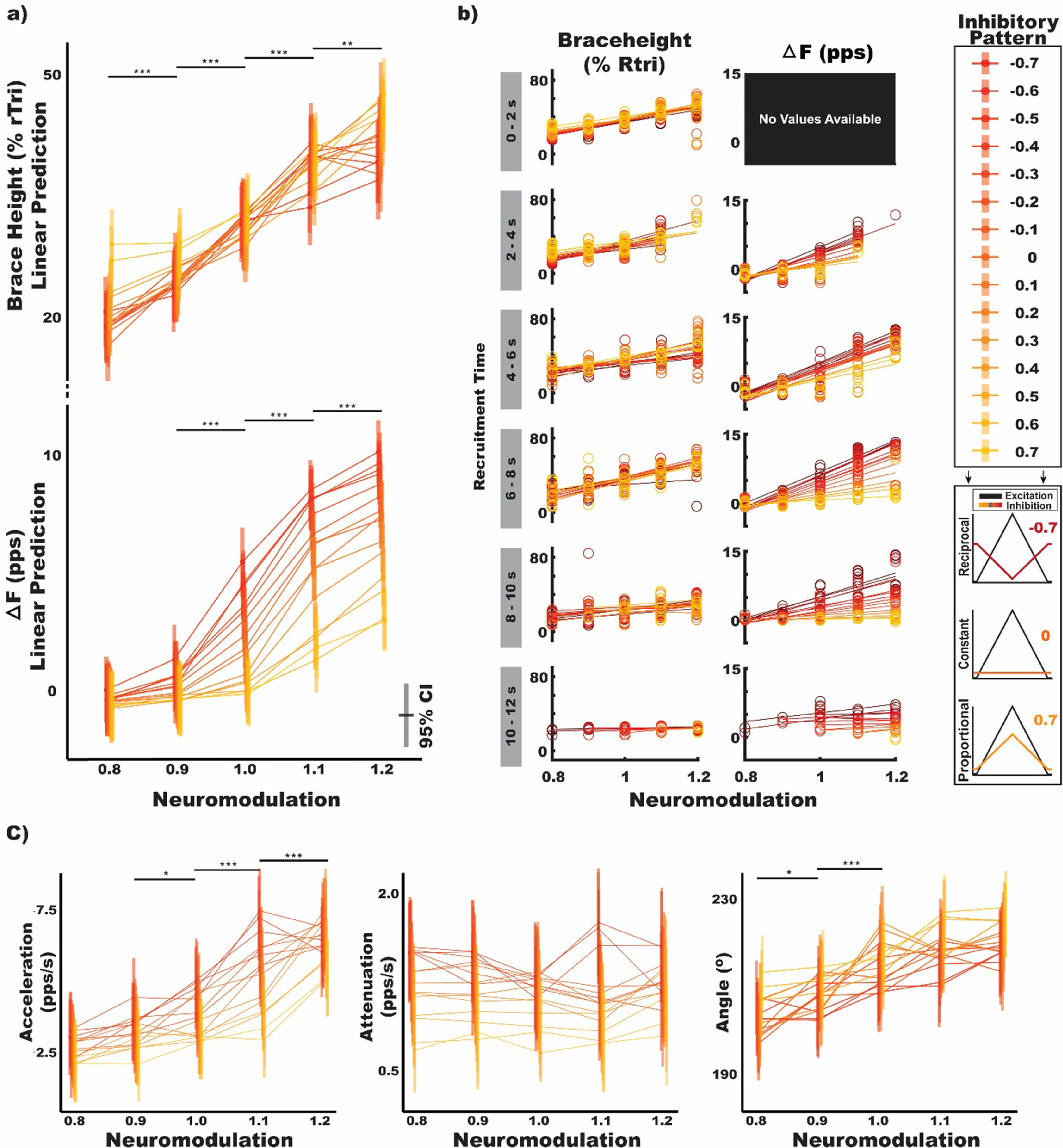
The effects of neuromodulation and inhibitory pattern on Δ*F*, brace height, acceleration slope, attenuation slope, and angle. Estimated values are shown on the specified scales with neuromodulation levels across the *x*-axis and vertical bars indicating mean and 95% confidence intervals (CI). The indicated neuromodulation values represent a multiplier applied to the max conductance of the L-type calcium channel used to simulate the PICs. Horizonal lines connect means for each level of inhibitory profile and are colored with reciprocal inhibitory patterns in red and proportional inhibitory patterns in yellow. The numerical values and color mapping indicated in the legend represent the excitation–inhibition coupling, with the pattern of inhibitory input varied in a reciprocal (−, red) or proportional (+, yellow) fashion. These values range from −0.7 to 0.7 and indicate the proportion of inhibition to excitation (i.e. 0.7: peak inhibition conductance ∼70% of excitation). For all panels, larger relative slopes indicate a strong effect of neuromodulation while a systematic spread of lines with different shades indicates a strong effect of the pattern of inhibition. Values in (a) and (c) represent model estimates for all simulated motoneurons whereas (b) represents motoneurons separated by the time at which they were recruited. When separated by recruitment, the lowest threshold group of MUs have no suitable Δ*F* values as there are no suitable reporter units at lower thresholds with adequate recruitment time differences required for full activation of the PIC (rTri: right triangle; pps: pulse-per-second; s: second).

To compare their ability for detecting potential changes in neuromodulatory or inhibitory inputs to MUs, we quantified brace height, its supplemental metrics, and Δ*F* for all MU discharge traces in the simulated motor pool. We then fit each of these metrics with a linear model that contained fixed effects of a MUs time of recruitment (0–2 s; 2–4 s; 4–6 s; 6–8 s; 8–10 s), neuromodulation (0.8, 0.9, 1.0, 1.1, 1.2), inhibition shape (−0.7, −0.6, −0.5, −0.4, −0.3, −0.2, −0.1, 0.0, 0.1, 0.2, 0.3, 0.4, 0.5, 0.6, 0.7), and their interaction. To observe the magnitude of change between either neuromodulation or inhibition patterns, we computed estimated marginal mean differences between factor levels. The progression of these relations across MUs of varying recruitment thresholds was further appreciated by segmenting the data along recruitment thresholds (0–2 s; 2–4 s; 4–6 s; 6–8 s; 8–10 s) as shown in figure 2(b). To provide a comparison of sensitivity between metrics, we computed standardized effect estimates by first normalizing each metric across the population of simulated MUs by subtracting the population mean and dividing by the population standard deviation. We then fit these populations with linear models as before and quantified the standardized effect estimates as the estimated marginal mean difference between sequential neuromodulation levels (0.8, 0.9, 1.0, 1.1, 1.2) or between the maximum and minimum inhibition profile (−0.7, 0.7). This allows us to observe the magnitude of difference between the estimated means for each level of neuromodulation and inhibitory pattern, in comparable units.

To compare the similarity of brace height, its supplemental metrics, and Δ*F* in human MUs, we quantified all metrics on the collected human dataset (number of spike trains, TA: 1448, MG: 2100, SOL: 1062, FDI: 2296). We fit a linear mixed effects model to each metric, consisting of muscle (TA, MG, SOL, FDI) and torque at recruitment (% MVT) as fixed effects and participant as a random effect. We then used estimated marginal means to compare the magnitude of difference between muscles for each metric. To observe the variability of each metric across repeated observations of the same unit, replicates of identical MUs were identified with a 2D cross correlation of MUAPs, as described, and the metrics for matched MUs were compared. To compare the variability, we quantified the coefficient of variation for each metric within a collection of matched MUs and predicted this with a linear mixed effect model consisting of metric (brace height, Δ*F*, acceleration slope, attenuation slope, angle) as a fixed effect and participant as a random effect.

All statistical analysis was performed with R (R Core Team, 2021). Mixed model analysis was achieved via the lme4 (Bats, Maechler, Bolker, & Walker, 2015) package and *p*-values were obtained by likelihood ratio tests of the full model with the effect in question against the model without the effect in question. For main effects, this included their subsequent interaction terms. To ensure the validity of model fitting, the assumptions of linearity and normal, homoscedastic residual distributions were confirmed. When appropriate, dependent variables were log-transformed and estimated marginal means back-transformed. Explicitly, predictions of acceleration slope were found to produce non-linear residuals and model predicted data that did not replicate the skewed distribution observed in acceleration slope and was remedied by log-transforming the dependent variable. Estimated marginal means were employed in pairwise post-hoc testing and achieved with the emmeans package (Lenth, 2022) and provided with 95% confidence intervals (95%CI). Estimated marginal means represent the means predicted from the statistical model for each relevant independent variable combination. They allow comparisons between the dependent variables of interest, while accounting for the appropriate fixed and/or random effects in the model. Significance was set at *α* = 0.05 and pairwise and multiple comparisons were corrected using Tukey’s corrections for multiple comparisons.

## 3. Results

### 3.1. Simulated MU findings

Using a simulated motor pool to isolate changes in neuromodulatory input and inhibitory pattern to motoneurons, the estimates for each metric across changes in neuromodulation and inhibition are shown in figure 2. As noted previously, changes in neuromodulatory drive were simulated by multiplying the max conductance of the L-type Calcium channel by a multiplier (0.8, 0.9, 1.0, 1.1, 1.2) to either increase or decrease the relative magnitude of PICs. In a physiological context, neuromodulation is generally associated with the level of monoaminergic drive (e.g. norepinephrine, serotonin) to the spinal cord. Greater levels of monoamines facilitate PICs, which provide a source of intrinsically driven excitatory current that alters the input–output relationship of the motoneuron. In a modeling context, we have defined neuromodulation as the conductance of the channels generating these PICs. Under the assumption that monoamines directly affect the magnitude of PICs, we are simulating the changes in PIC properties that would be expected with changes in monoamines. Similarly, we simulated changes in the pattern of inhibition by adjusting the dendritic inhibitory conductance as a function of the excitatory synaptic inputs. Specifically, inhibitory conductance was adjusted to recreate an inhibitory input that positively scaled with the excitatory input (proportional) or negatively scaled with excitatory input (reciprocal). (Johnson *et al* 2017) This excitation–inhibition coupling can be appreciated in figure 2 with values reported from −0.7 to 0.7 and corresponding to reciprocal and proportional relationships, respectively, with the value indicating the proportion of inhibition to excitation (i.e. 0.7: peak inhibition conductance ∼70% of excitation).

In total, this simulation includes 1500 discharge profiles from 20 simulated motoneurons at each possible combination of 15 inhibition levels and 5 neuromodulation levels. Given the criteria for inclusion with each metric, this provided 846 estimates for Δ*F* and 1415 for brace height, acceleration slope, attenuation slope, and angle. In figure 2, the varying shades (yellow–red) indicate the inhibitory pattern and are connected across the levels of neuromodulation indicated on the *x*-axis. Larger relative changes traversing the *x*-axis indicate greater sensitivity to neuromodulation (figure 2(a), brace height and Δ*F*), while tighter spacing along the *y*-axis indicates lower sensitivity to varying inhibitory patterns (figure 2(a), brace height).

Across sequentially greater levels of simulated neuromodulation, we observed brace height to increase similarly for each level of inhibition (figure 2(a)) and found neuromodulation to be a significant predictor of this value (*F* = 236.31, *p* < 0.001). As detailed in section 2, values of brace height are normalized and provided as a percentage of the brace height for a right triangle. Averaging across levels of inhibition, brace height is estimated to significantly increase by 4.67 (95%CI: [2.80, 6.53]), 6.41 (95%CI: [4.59, 8.23]), 8.55 (95%CI: [6.70, 10.39]), and 5.37 (95%CI: [3.41, 7.32]) for each increase in neuromodulation level from 0.8–0.9, 0.9–1.0, 1.0–1.1, and 1.1–1.2, respectively. Conversely, though we found the inhibitory pattern to be a significant predictor of brace height (*F* = 3.00, *p* = 0.001), post-hoc testing reveals no significant differences between sequential increases in inhibitory pattern. Additionally, brace height appears insensitive to relatively large changes in inhibitory pattern, with estimates of brace height not significantly different from zero between strong reciprocal inhibition (−0.7) and constant inhibition (0) (−1.75; 95%CI: [−5.41, 1.90]), or between constant inhibition (0) and strong proportional inhibition (0.7) (−3.30; 95%CI: [−6.91, 0.31]).

Similar to brace height, we found neuromodulation to be a significant predictor of Δ*F* (*F* = 330.66, *p* < 0.001). In contrast to brace height, we found that neuromodulation was not a significant predictor of Δ*F* changes at lower levels of neuromodulation (0.52 pps; 95%CI: [−0.05, 1.09]), whereas, at higher levels of neuromodulation, Δ*F* is estimated to significantly increase by 1.75 pps (95%CI: [1.23, 2.26]), 3.85 pps (95%CI: [3.38, 4.33]), and 2.47 pps (95%CI: [1.99, 2.94]) for each increase in neuromodulation level from 0.9–1.0, 1.0–1.1, and 1.1–1.2, respectively. Interestingly, Δ*F* exhibits an interaction between neuromodulation and inhibitory shape (*F* = 14.06, *p* < 0.001) with differences in Δ*F* between neuromodulation levels greater under reciprocal inhibition (figure 2(a)). Likely as a product of this interaction, Δ*F* displays a dependence on changes in inhibitory pattern (*F* = 135.21, *p* < 0.001). Averaging across levels of neuromodulation, we found Δ*F* to significantly decrease by 1.96 pps (95%CI: [1.02, 2.91]) between strong reciprocal inhibition (−0.7) and constant inhibition (0), and by 2.39 pps (95%CI: [1.35, 3.44]) between constant inhibition (0) and strong proportional inhibition (0.7).

Separating Δ*F* and brace height estimates into cohorts based on their recruitment threshold (figure 2(b)) allows their dependence on neuromodulation and inhibition to be further appreciated. Across recruitment threshold groups, values of brace height display a similar dependence on neuromodulation for all inhibition patterns, while Δ*F* displays an apparent interaction between inhibition and neuromodulation. Of note, the lowest threshold group of MUs displays no suitable Δ*F* values because there are no suitable reporter units, an inherent problem of this paired unit analysis for estimating the effects of PICs on low threshold units.

For the additional metrics garnered from brace height quantification (see section 2 and figure 2(c)), we found neuromodulation to be a significant predictor of acceleration slope (*F* = 68.90, *p* < 0.001) and angle (*F* = 39.74, *p* < 0.001), but not attenuation slope (*F* = 0.93, *p* = 0.44). Acceleration slope, averaging across levels of inhibition, is estimated to significantly increase by 0.67 pps s^−1^ (95%CI: [0.04, 1.30]), 1.43 pps s^−1^ (95%CI: [0.78, 2.07]), and 1.15 pps s^−1^ (95%CI: [0.49, 1.81]) for increases in neuromodulation level from 0.9–1.0, 1.0–1.1, and 1.1–1.2, respectively. Conversely, angle is estimated to significantly increase by 4.60° (95%CI: [0.79, 8.40]) and 6.55° (95%CI: [2.70, 10.41]) for increases in neuromodulation level from 0.8–0.9 and 0.9–1.0, respectively.

We found inhibition pattern to be a significant predictor of attenuation slope (*F* = 67.96, *p* < 0.001), acceleration slope (*F* = 4.15, *p* < 0.001), and angle (*F* = 2.78, *p* < 0.001). Attenuation slope is estimated to significantly increase by 0.50 pps s^−1^ (95%CI: [0.18, 0.82]) from proportional inhibition (0.7) to constant inhibition (0), with no significant changes from strong reciprocal (−0.7) to constant (0) but an estimated decrease of 0.71 pps s^−1^ (95%CI: [0.38, 1.04]) from strong reciprocal (−0.7) to strong proportional (0.7). We found acceleration slope to significantly increase by 0.32 pps s^−1^ (95%CI: [0.09, 0.56]) from proportional inhibition (0.7) to constant inhibition (0), with no significant changes from strong reciprocal (−0.7) to constant (0) but an estimated decrease of 0.37 pps s^−1^ (95%CI: [0.14, 0.62]) from strong reciprocal (−0.7) to strong proportional (0.7). Similarly, we found angle to significantly increase by 7.03° (95%CI: [0.69, 13.37]) from proportional inhibition (0.7) to constant inhibition (0), with no significant changes from strong reciprocal (−0.7) to constant (0) but an estimated decrease of 9.39° (95%CI: [3.27, 16.33]) from strong reciprocal (−0.7) to strong proportional (0.7).

**Figure 3.**
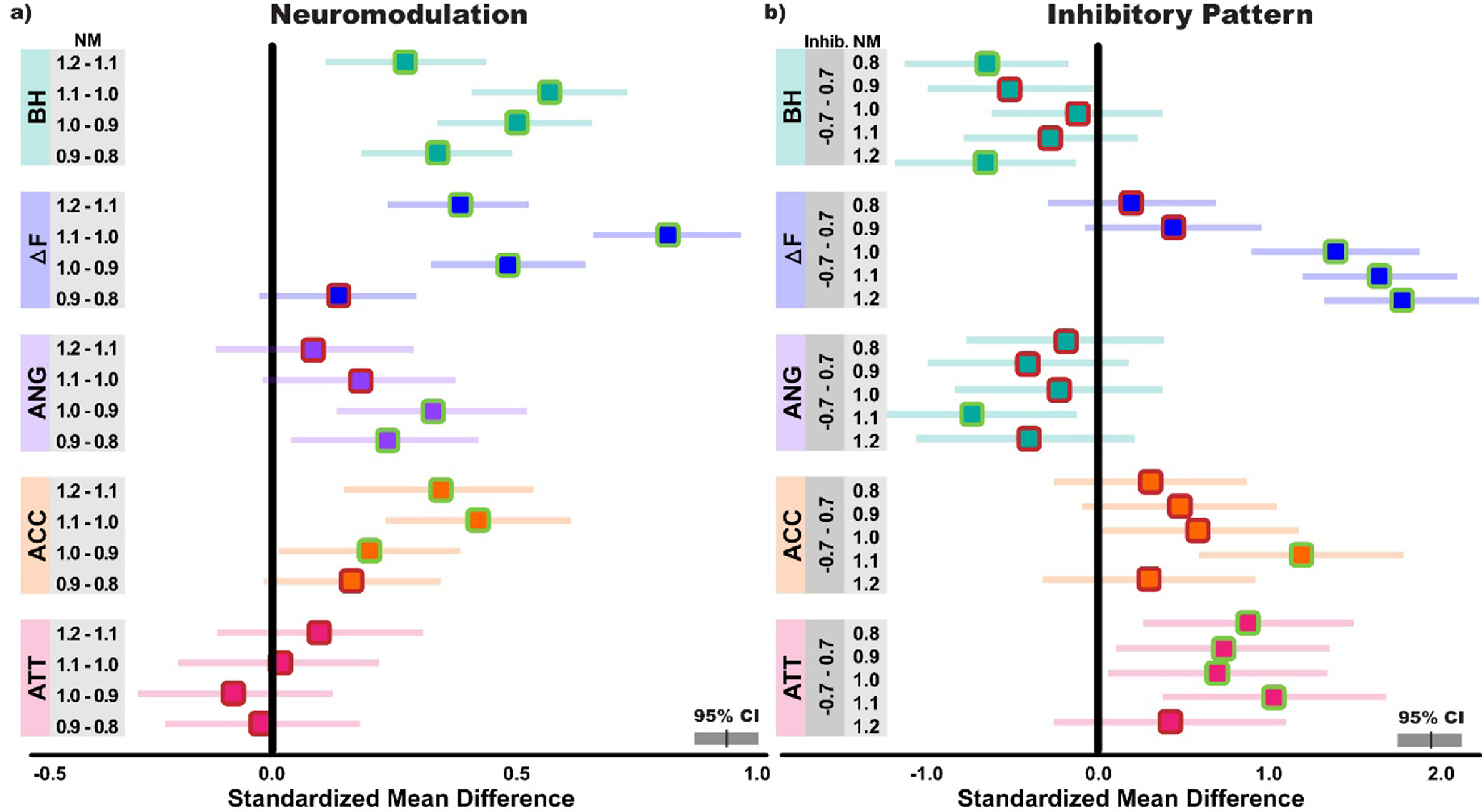
The standardized effects of neuromodulation and inhibitory pattern on Δ*F*, brace height, acceleration slope, attenuation slope, and angle. Values are shown on comparable scales, divided by the population standard deviation for each metric, and represent the standardized mean differences between each indicated level of neuromodulation or inhibitory pattern. This provides an estimate of the magnitude of change for each metric relative to the variance of that metric, where larger values indicate a greater effect. Estimates on the left (a) indicate the predicted change in metric between the indicated neuromodulation (NM) level while estimates on the right (b) indicate the predicted change in metric between strong proportional (0.7) and reciprocal (−0.7) inhibition at the indicated neuromodulation level. Standardized mean differences are displayed with 95% confidence intervals (CI), with means highlighted in green when excluding zero (BH: brace height; ANG: angle; ACC: acceleration slope; ATT: attenuation slope).

Supporting these estimates of absolute differences, standardized mean differences can be appreciated for sequential changes in neuromodulation in figure 3(a) and maximal changes in inhibition shape (−0.7–0.7) in figure 3(b). Since the raw values for each metric are on differing scales, figure 3 presents the magnitude of these differences on comparable scales and represents the estimated mean difference as a function of standard deviation. Larger values indicate a greater effect of the change in neuromodulation or inhibition represented in the grey boxes, with the sign of the change indicating the direction. For changes in neuromodulation, these standardized estimates track the absolute differences, with significant findings paralleling those previously reported, but with their magnitude indicated on similar scales. When looking at maximal changes in inhibition pattern (reciprocal–proportional), brace height is estimated as significantly different at only the highest and lowest neuromodulation levels (0.8, 1.2), Δ*F* is significantly different at neuromodulation levels of 1.0, 1.1, and 1.2, acceleration slope and angle appear relatively insensitive to large changes in inhibition, and attenuation slope is significantly different for all but the highest neuromodulation level.

### 3.2. Human MU findings

#### 3.2.1. All MUs

**Figure 4.**
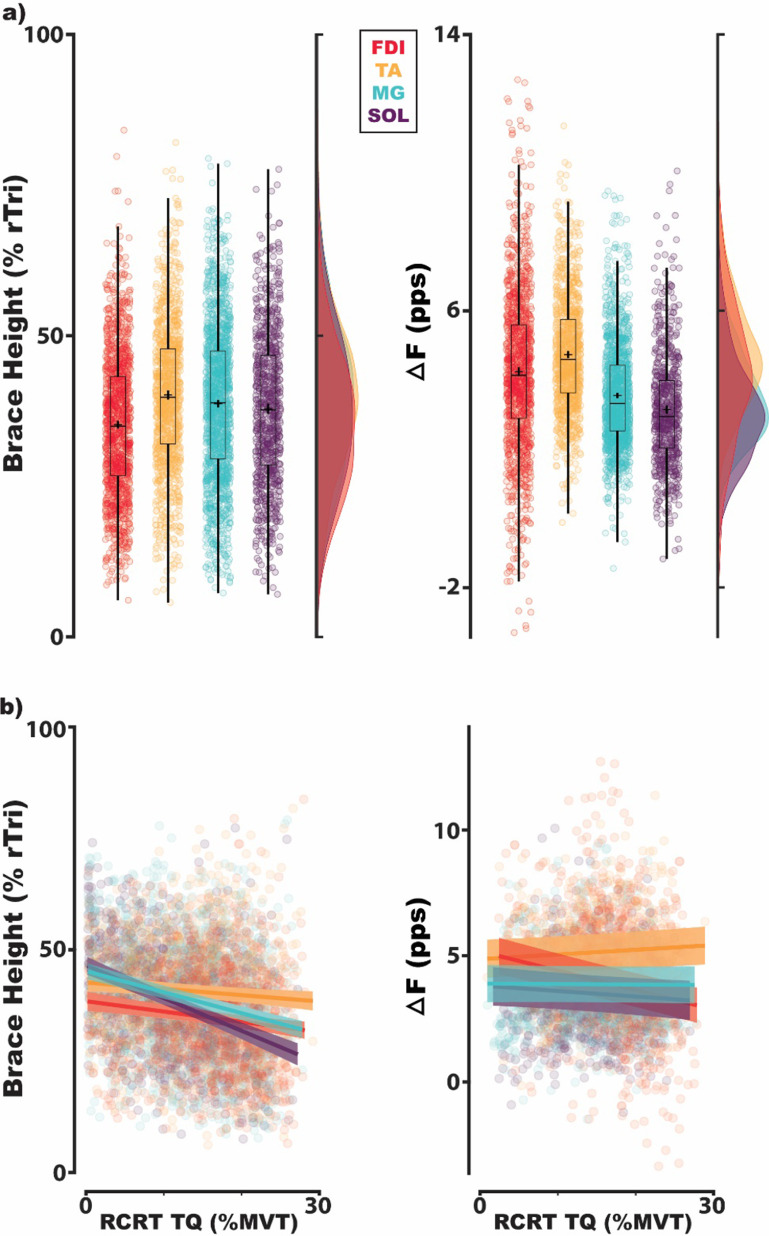
Estimates of brace height and Δ*F* for MUs of human muscles in the distal upper and lower limb. This includes the tibialis anterior (TA), medial gastrocnemius (MG), soleus (SOL), and first dorsal interosseus (FDI). Each data point indicates estimates for a single MU and is colored by its respective muscle. For estimates collapsed across torque at recruitment (a), traditional box plots overly the population of MUs, with estimated means indicated by a cross and corresponding probability density generated with a Gaussian kernel. Values in (b) show all MUs as a function of the torque at which a MU was recruited with muscles similarly indicated by color (rTri: right triangle; pps: pulse-per-second; RCRT TQ: torque at recruitment; MVT: maximum voluntary torque).

**Figure 5.**
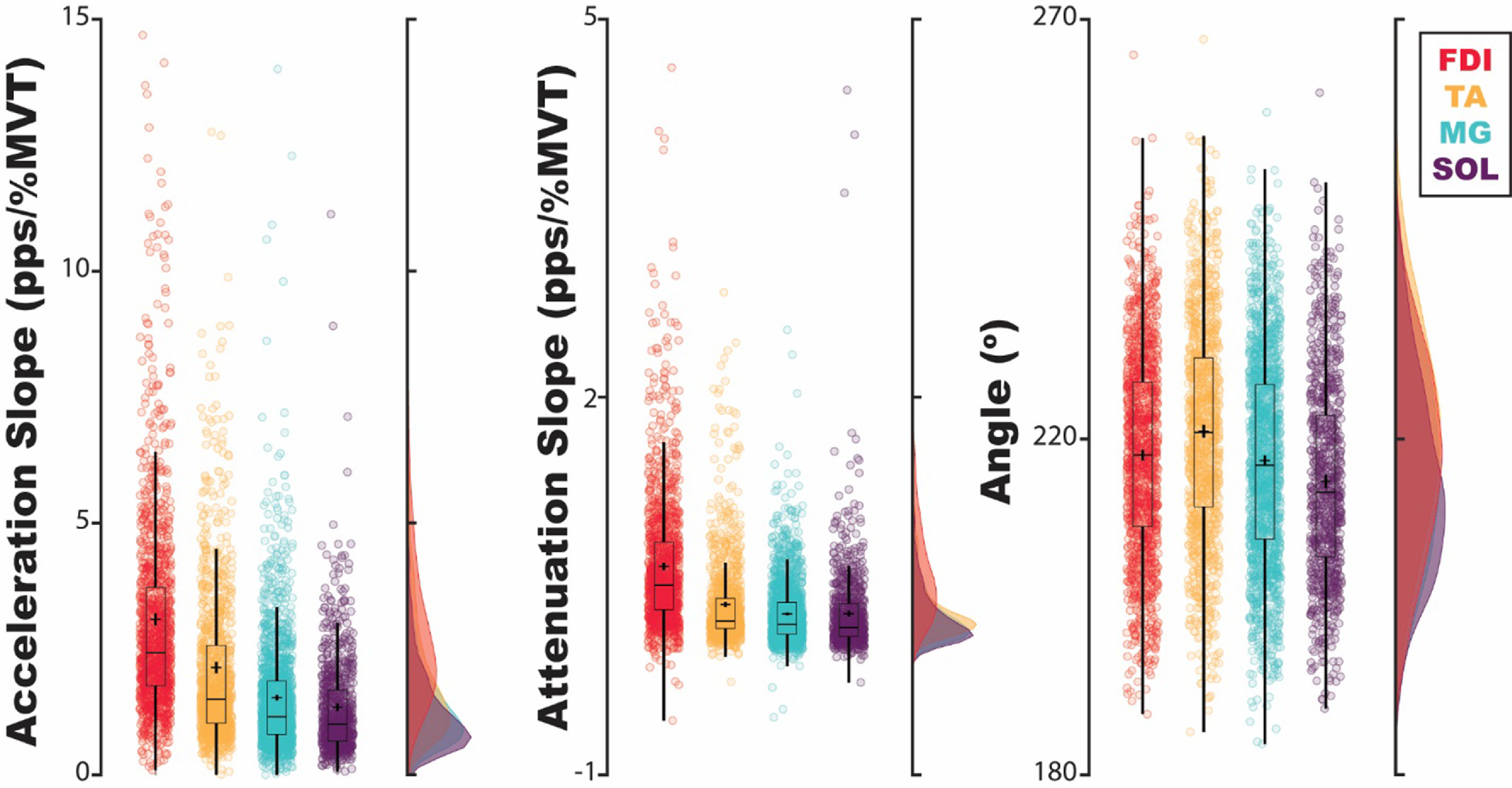
Estimates of acceleration slope, attenuation slope, and angle for MUs of human muscles in the distal upper and lower limb. This includes the tibialis anterior (TA), medial gastrocnemius (MG), soleus (SOL), and first dorsal interosseus (FDI). Each data point indicates estimates for a single MU and is colored by its respective muscle. Traditional box plots overly the population of MUs, with estimated means indicated by a cross and corresponding probability density generated by a Gaussian kernel (pps: pulse-per-second; MVT: maximum voluntary torque; s: second).

To observe the behavior of brace height, its supplemental metrics, and Δ*F* in human MUs, we quantified all metrics on a collection of human MUs (TA: 1448, MG: 2100, SOL: 1062, FDI: 2296). These values can be seen for Δ*F* and brace height in figure 4(a) and acceleration slope, attenuation slope, and angle in figure 5. Owing to the inclusion criteria employed for each metric, this included fewer estimates for Δ*F* (TA: 1071, MG: 1484, SOL: 701, FDI: 1284) than for brace height, acceleration slope, attenuation slope, and angle (TA: 1150, MG: 1676, SOL: 848, FDI: 1359).

For both Δ*F* and normalized brace height, we found muscle (Δ*F*: [*χ* ^2^(3) = 386.08, *p* < 0.001]; brace height: [*χ* ^2^(3) = 77.06, *p* < 0.001]), torque at MU recruitment (Δ*F*: [*χ* ^2^(1) = 7.62, *p* = 0.006]; brace height: [*χ* ^2^(1) = 204.34, *p* < 0.001]), and their interaction (Δ*F*: [*χ* ^2^(3) = 76.26, *p* < 0.001]; brace height: [*χ* ^2^(3) = 67.19, *p* < 0.001]) to be significant predictors. Estimated marginal means of Δ*F* and brace height for each muscle are shown in table 1 averaged across recruitment thresholds. Post-hoc analysis indicates estimates of brace height to be significantly different between all muscles except FDI and SOL while Δ*F* is estimated to be significantly different between all but the FDI and TA. Expanding estimates across the recruitment threshold of MUs, distinct relationships can be observed for Δ*F* and brace height. As seen in figure 4(b), across all muscles, Δ*F* and brace height decrease with MUs recruited at higher levels of torque. Traversing from 1% MVT to 30% MVT, averaging across muscles, Δ*F* is estimated to decrease by 0.018 pps (Cohen’s *d*: 0.0015 [0.0001, 0.0024]) and brace height is estimated to decrease by 0.40% rTri (Cohen’s *d*: 0.033 [0.023, 0.044]). Though significant differences are observed between muscles and across recruitment threshold, the effects are noticeably small and may possess no functional relevance.

**Table 1.** Average estimates of Δ*F*, brace height, acceleration slope, attenuation slope, and angle for each muscle (TA: tibialis anterior; MG: medial gastrocnemius; SOL: soleus; FDI: first dorsal interosseus). Estimates represent marginal means and are reported as mean and 95% confidence interval (*μ* [95%CI]) (rTri: right triangle; pps: pulse-per-second; MVT: maximum voluntary torque; s: second).

	Estimated metric values			
	MG	TA	SOL	FDI
Δ*F* (pps)	3.77 [3.30, 4.25]	5.06 [4.58, 5.54]	3.43 [2.95, 3.91]	4.80 [4.31, 5.30]
Brace height (% rTri)	38.4 [37.0, 39.7]	40.7 [39.3, 42.1]	35.7 [34.2, 37.2]	34.5 [32.9, 36.0]
Acceleration slope (pps/MVT)	1.79 [1.54, 2.04]	2.44 [2.18, 2.70]	1.66 [1.40, 1.93]	3.12 [2.85, 3.39]
Attenuation slope (pps/MVT)	0.329 [0.290, 0.367]	0.412 [0.372, 0.452]	0.384 [0.341, 0.427]	0.624 [0.579, 0.669]
Angle (°)	218 [217, 220]	222 [220, 223]	216 [214, 217]	218 [216, 220]

For both acceleration and attenuation slope, we found muscle to fail as a significant predictor but torque at recruitment (acceleration: [*χ* ^2^(1) = 613.64, *p* < 0.001]; attenuation: [*χ* ^2^(1) = 988.52, *p* < 0.001]) and their interaction (acceleration: [*χ* ^2^(3) = 69.29, *p* < 0.001]; attenuation: [*χ* ^2^(3) = 67.83, *p* < 0.001]) to be significant predictors. In contrast, for angle, we found recruitment threshold to fail as a significant predictor but muscle (*χ* ^2^(3) = 127.32, *p* < 0.001) and their interaction (*χ* ^2^(3) = 49.22, *p* < 0.001) to be significant predictors. Marginal means for each metric and each muscle averaged across recruitment thresholds can be seen in table 1.

#### 3.2.2. Matched MUs

**Figure 6.**
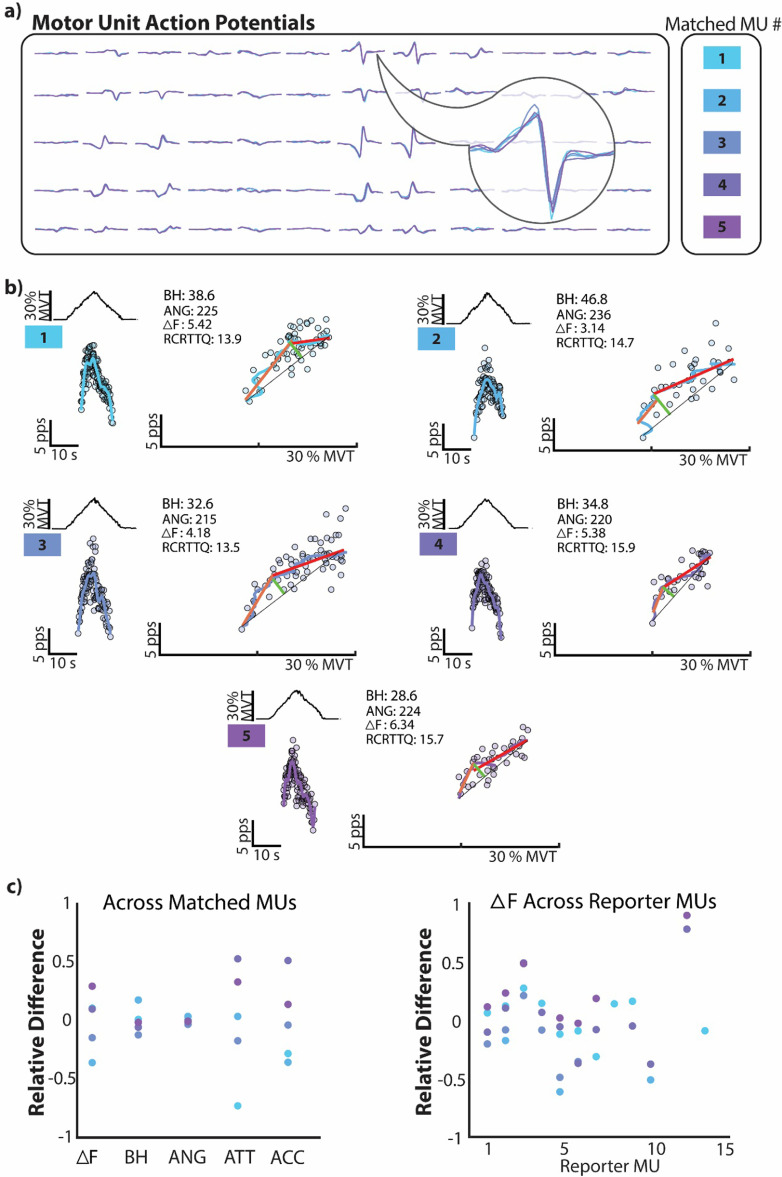
A single tibialis anterior motor unit (MU), matched across five independent triangular contractions. The overlying motor unit action potential waveforms (MUAPs) are shown in (a) and organized according to the electrode grid used in data collection. Each of the five instances of this unique MU are shown in (b) with the dorsiflexion torque for the given trial in the top left, the MUs discharge rate as a function of time in the bottom left, and the brace height quantification on the right. Additionally, values for brace height (BH), angle (ANG), Δ*F*, and torque at recruitment (RCRT TQ) are shown for each matched MU. For each repeated instance of this MU, the relative difference is shown as a function of the mean values for each metric (c). This relative difference was quantified as the absolute deviation of a given replicate from the group mean for all replicates of a unique unit, in terms of the group mean. Additionally, given that the presented Δ*F* for each test MU represents the average Δ*F* across all suitable lower threshold reporter MUs, these relative differences are shown for all Δ*F* test unit–reporter unit pairs on the bottom right (c). The relative mean difference allows one to observe the dispersion of estimates for each observation around the population average, on comparable scales for each metric (BH: brace height; ANG: angle; ACC: acceleration slope; ATT: attenuation slope; pps: pulse-per-second, MVT: maximum voluntary torque, s: second).

To observe the variability of each metric across repeated observations of the same unit, replicates of identical TA MUs were identified in repeated ramp contractions, as described, yielding a total of 158 unique MUs with an average of 3.30 (SD: 1.63) occurrences. An example of a single unique MU can be seen in figure 6, with each metric quantified for all five of its occurrences in figure 6(b) and its MUAPs in figure 6(a). The relative difference from group average for each metric across this MU’s five observances are shown on the left in figure 6(c) and provide insight into relative variability, or the dispersion of estimates for each observation around the population average. For each replicate, this relative difference was quantified as the absolute deviation of a given replicate from the group mean for all replicates of a unique unit, in terms of the group mean. Using the relative mean difference allows for the variation of the estimated values to be observed on comparable scales and makes no underlying assumption on the distribution of the data or the presence of outliers. Furthermore, given that we have quantified Δ*F* for each test unit as the average value across all reporter MUs (see section 2), the relative differences between all pairwise Δ*F* values (i.e. each test unit–reporter unit pair) is shown on the right of figure 6(c). Averaging across reporter units for each test unit reduces the inherent variability of Δ*F* values, which can be observed with the dispersion of values across reporter units in the right plot of figure 6(c).

Across 158 unique MUs, 135 MUs yielded valid estimates for Δ*F* while 142 MUs were estimated with brace height and its associated metrics. This created an average number of repeated unit observations of 2.34 (SD: 1.82) for Δ*F* and 2.91 (SD: 1.74) for brace height. Fitting a linear mixed model to the coefficient of variation of metric estimates for replicates of each unique MU, we found the type of metric to be a significant predictor (*χ* ^2^(5) = 373, *p* < 0.001). Marginal means for the coefficient of variation are estimated as 0.120 (95%CI: [0.0924, 0.155]) for Δ*F*, 0.138 (95%CI: [0.108, 0.177]) for brace height, 0.318 (95%CI: [0.248, 0.408]) for acceleration slope, 0.245 (95%CI: [0.191, 0.314]) for attenuation slope, 0.0277 (95%CI: [0.0216, 0.0355]) for angle, and 0.129 (95%CI: [0.101, 0.166]) for torque at recruitment. Of note, all pairwise comparisons between metric estimates are predicted significantly different (*p* < 0.0001), except the differences between Δ*F* and brace height, Δ*F* and torque at recruitment, brace height and torque at recruitment, and between acceleration and attenuation slope.

## 4. Discussion

As a result of the high fidelity of action potentials within a MU, their discharge patterns provide a unique window into the composition of excitatory, inhibitory, and neuromodulatory commands to motoneurons. In the present study, we present a complementary approach to the most commonly used methods for quantifying the contributions of these commands to the discharge profile of human MUs. In particular, we propose quantifying discharge rate nonlinearity following the onset of MU recruitment during time varying linear tasks, as this nonlinearity is commonly attributed to neuromodulatory commands and believed indicative of intrinsic activation by monoaminergic dependent PICs (i.e. PIC amplification). We term this nonlinearity brace height and use its instance of occurrence to garner additional metrics (i.e. acceleration slope, attenuation slope, and angle) for characterizing the secondary and tertiary range of MU discharge. We then compare these metrics to the commonly used paired MU analysis (Δ*F*) on a collection of human MUs and investigate their ability to detect changes in neuromodulatory and inhibitory drive to a simulated pool of motoneurons with known inputs.

### 4.1. Detection of known inputs to simulated motoneurons

Using a simulated motoneuron pool, we found brace height and its associated metrics to provide insight into the organization of neuromodulatory and inhibitory inputs to motoneurons that are complementary to Δ*F*. In total, of the 1500 simulated discharge traces, Δ*F* produced 846 viable estimates, while brace height, acceleration slope, attenuation slope, and angle each produced 1415 estimates. Additionally, given that Δ*F* employs a paired analysis scheme where each estimate is based upon values obtained in lower threshold reporter motoneurons, lower threshold motoneurons were less likely to yield an estimate. This creates a situation where Δ*F* estimates are necessarily confined to relatively higher threshold MUs, as can be seen in figure 2(b) where no suitable Δ*F* pairs were found for motoneurons recruited 0–2 s. Conversely, brace height provides estimates on a single unit level and does not require the pairing of units across recruitment thresholds. In addition to the greater yield of valid estimates, the single unit estimates accomplished with brace height may allow for robust estimates of neuromodulatory drive.

#### 4.1.1. Neuromodulation

Across all reported metrics, sequential increases in neuromodulatory drive appear to be most consistently represented with brace height (figure 3) in the simulated motor pool. On comparable scales (standardized mean differences), brace height yields an average estimated change of 0.419 (SD: 0.137) across sequential increases of 0.1 in neuromodulation (0.8, 0.9, 1.0, 1.1, 1.2), while Δ*F* produces an estimated average change of 0.452 (SD: 0.280). Though similar in magnitude, the greater consistency of brace height across neuromodulation levels can be appreciated in figures 2 and 3, where Δ*F* displays a wider range of sensitivity between neuromodulation values and proves insensitive to neuromodulation changes between the lower spectrum of values (0.8–0.9). Similarly, values of acceleration slope and angle show inconsistent sensitivity to changes in neuromodulation, with no predicted changes for acceleration slope at lower values of neuromodulation and for angle at higher values of neuromodulation (figures 2 and 3).

The sensitivity of the brace height measure to neuromodulatory drive is likely a consequence of its direct quantification of deviation from linearity in the ascending phase of motoneuron discharge. During a linear increase in effort, this deviation is thought to be a result of intrinsic activation from PICs, and due to the facilitation of PICs by monoamines, should be an ideal proxy for neuromodulatory drive. As previously discussed, this is supported by prior work that shows PICs to amplify excitatory synaptic input as a function of monoaminergic drive (Hounsgaard and Kiehn 1985, Crone *et al* 1988, Hounsgaard *et al* 1988, Heckman *et al* 2008). Nevertheless, brace height may better indicate the contributions of Na PICs to the discharge of a motoneuron, as opposed to Ca PICs. In general, mammalian motoneurons exhibit PICs generated by both L-type Ca2+ channels (CaPIC) and persistent NA+ channels (NaPIC) (Lee and Heckman 1998b, Li *et al* 2004, Heckman and Enoka 2012). The CaPIC has a longer time course, is readily persistent, and is likely responsible for discharge rate hysteresis (Svirskis and Hounsgaard 1997, Lee and Heckman 1998a, Li and Bennett 2003, Elbasiouny *et al* 2006, Moritz *et al* 2007). In contrast, the NaPIC is rapid in both activation and inactivation and generates inwards currents over a shorter duration that are essential in spike initiation during repetitive discharge (Lee and Heckman 1999b, 2001, Kuo *et al* 2006, Harvey *et al* 2006b). As a result of the faster action of the NaPIC, and the insensitivity of brace height to persistent discharge, the normalized brace height value we present may possess a greater contribution of the NaPIC than captured with Δ*F*.

Although dependent on neuromodulatory drive in these simulations, brace height is likely limited in the aspects of PICs and neuromodulatory drive that it can detect. Indeed, as noted, brace height is likely impervious to discharge rate hysteresis, which Δ*F* readily quantifies. That said, the quantification of Δ*F* is also dependent on how accurately lower threshold reporter units represent synaptic drive to the motor pool. Any alterations in lower threshold MUs that change the ascending or descending phase of discharge (e.g. inhibitory input) could also affect Δ*F* estimates and may confound interpretations of hysteresis. Specifically, given that units are paired across recruitment thresholds to obtain Δ*F* estimates, changes in Δ*F* cannot be isolated to alterations in either the discharge profile of the reporter unit or hysteresis of the test unit, making interpretations across recruitment threshold difficult. To overcome this potential limitation, we have employed a test-unit average scheme and various control conditions to find suitable reporter units (see section 2). With this in mind, although brace height quantification does provide single unit estimates and can provide detailed insight into changes across recruitment threshold, it does not provide direct quantification of hysteresis and thus further work should be directed on this front.

#### 4.1.2. Inhibition pattern

Controlling the pattern of inhibitory input to the pool of simulated motoneurons, we found attenuation slope to be most consistently predicted by the shape of inhibitory commands. In specific, we found values of attenuation slope to be significantly different between strong reciprocal (−0.7) to strong proportional inhibition (0.7) in four of the five neuromodulation levels (0.8, 0.9, 1.0, 1.1). Averaging across all neuromodulation levels yields a standardized mean difference of 0.721 (95%CI95%CI: [0.433, 1.01]) from strong reciprocal to proportional inhibition. It is speculated that inhibitory-driven changes in rate modulation are responsible for these observed differences, as suggested previously (Powers *et al* 2012, Powers and Heckman 2017). In contrast, brace height displayed significant differences only at extreme neuromodulation levels (0.8, 1.2) while acceleration slope and angle were significantly different at a single neuromodulation level (1.1), making these metrics unlikely candidates for quantifying changes in inhibitory command profile.

In addition to changes in attenuation slope, altering the pattern of inhibitory input revealed a distinct interaction between inhibition and neuromodulation in Δ*F*. This interaction can be readily observed in figures 2 and 3, with changes in Δ*F* from inhibition greatest at higher neuromodulation levels. Averaging across all neuromodulation levels yields a standardized mean difference of 1.06 (95%CI: [0.844, 1.27]) from strong reciprocal (−0.7) to proportional inhibition (0.7). That said, Δ*F* appears to be largely insensitive to changes in neuromodulation and inhibition profile when neuromodulation values are lower. This may be due to the bias inhibition employed in our simulations. A constant bias inhibitory offset to the motoneuron pool was employed in all simulations and is necessary to ensure the simulated motoneurons cease discharge at the cessation of excitatory input. As Δ*F* quantifies discharge rate hysteresis, the employed neuromodulation parameters were likely insufficient to generate any appreciable hysteresis at lower values and thus produced the observed floor effect. Regardless, a significant interaction is still observed and implies that changes in Δ*F* are greatest at both higher levels of neuromodulation and greater reciprocal inhibition (−0.7), and are attenuated under proportional inhibition (0.7) and at lower neuromodulation levels.

#### 4.1.3. *Brace height and* ΔF

In total, although Δ*F* and brace height respond similarly to changes in the neuromodulatory commands to motoneurons, they differ in their response to changes in inhibitory commands, thus providing complementary information. The absence of a strong relation to the pattern of inhibitory input may allow brace height to be a more focal quantifier of neuromodulatory commands and allow for inhibitory and neuromodulatory driven changes in Δ*F* to be decoupled. Furthermore, the additional metrics achieved with brace height quantification may assist in this decoupling process and provide a more granular view of changes in MU discharge patterns. Specifically, as indicated by the simulation results, the attenuation slope appears to be a consistent quantifier of the pattern of inhibitory input while the acceleration slope and angle may additionally indicate changes in neuromodulation. Exploiting these relations and using brace height and attenuation slope to estimate neuromodulatory and inhibitory commands, respectively, may provide a greater potential for understanding the synaptic organization of motor commands to the motor pool.

### 4.2. Comparison of metrics in human MUs

Estimated average Δ*F*, brace height, acceleration slope, attenuation slope, and angle values can be seen for each muscle in table 1. These estimates indicate normative values for each muscle and can be further appreciated in figures 4 and 5. Of the included collection of human MUs (TA: 1448, MG: 2100, SOL: 1062, FDI: 2296), the inclusion criteria necessary for Δ*F* generated reduced estimates (TA: 1071, MG: 1484, SOL: 701, FDI: 1284) when compared to brace height, attenuation slope, acceleration slope, and angle (TA: 1150, MG: 1676, SOL: 848, FDI: 1359).

For both Δ*F* and brace height, we found muscle to be a significant predictor, with brace height significantly different between all muscles except FDI and SOL and Δ*F* significantly different between all but FDI and TA. Of note, though estimated as significantly different, the distributions indicated in figure 4(a) appear less separable between muscles for brace height. This is likely due to the relationship between brace height and the recruitment threshold of MUs. Compared to Δ*F*, we found a greater relative decrease in brace height with increasing values of recruitment threshold (figure 4(b)). This greater relative range likely accounts for the wider distributions when collapsed across all observed MUs and was accounted for in our marginal estimates.

Comparing angle, acceleration slope, and attenuation slope across muscles, angle parallels trends observed with brace height while the slope parameters require a more nuanced interpretation. For all muscles, the slope estimates in both regions appear to covary with one another but diverge from the trends observed with brace height. Qualitatively, we observed the FDI to generate the greatest slopes, followed by the TA and then the MG and SOL. Interestingly, though the FDI and TA possess greater acceleration and attenuation slopes, the FDI and TA produce the smallest and largest brace height values, respectively. Though many factors may contribute to these estimates (e.g. starting discharge rate, peak discharge rate) this observation can likely be attributed to a greater acceleration slope relative to attenuation slope in the TA (i.e. acceleration/attenuation slope ratio). In general, we theorize that a greater acceleration slope would indicate a MU that has quickly turned on, likely as a result of intrinsic activation from PICs (Lee and Heckman 2000, Johnson *et al* 2017, Powers and Heckman 2017). Conversely, a MU that exhibits both a high acceleration and attenuation slope may indicate a more linear increase to peak discharge and little intrinsic activation from PICs. Indeed, a similar rationale is employed by those who fit rising exponential functions to the ascending phases of MU discharge (Revill and Fuglevand 2017, Kirk *et al* 2021, Zero *et al* 2022).

Despite these significant differences between muscles, the estimated effect sizes are notably small and thus predicted neuromodulation and inhibitory patterns for each muscle would likely be similar. While first speculation may bias toward expectations of variance in neuromodulation or inhibitory patterns amongst muscles, as discharge patterns are noticeably different, the pattern of descending neuromodulatory projections suggests a more uniform effect. Monoaminergic drive, which facilitates PICs, is largely diffuse in nature with output that spans the spinal cord (Bowker *et al* 1982, Holstege and Kuypers 1987). Such a diffuse output likely affects varying muscles in an equal manner. Furthermore, though spinal circuitry yields potential mechanisms for adjusting inhibitory patterns to muscles independently, the estimates here appear to imply the muscles in this present study are similarly under only a mild form of inhibition.

### 4.3. Consistency of measures across repeated observations

Investigating the variation in estimates across repeated observations of the same MU, we found brace height and Δ*F* to provide comparable variability. This can be observed in a choice example of five replicates of the same MU in figure 6. In this example, estimates of Δ*F* and brace height show comparable variability, angle displays low variability, and both slope parameters appear highly variable (figures 6(b) and (c)). Of note, though Δ*F* provides estimates that are all within ±50% of the group mean, as seen with the relative mean difference in figure 6(c), this may be an artifact introduced by the test unit averaging methods employed. For the quantification of Δ*F* in this study, we estimated Δ*F* for each test unit as the average Δ*F* across all reporter unit pairs. This necessarily reduces the inherent variability of Δ*F*, which can be seen in the values of relative mean differences across reporter MUs in the right plot of figure 6(c). In this rightmost plot, the dispersion of values across reporter unit pairs appears greater and is likely due to variations in the behavior among the reporter units (lower threshold MUs).

Comparing this variability systematically across all unique MUs, we found brace height and Δ*F* to display similar values of coefficient of variation for repeated observations. Of the 158 unique MUs, this included 135 MUs with an average of 2.34 (SD: 1.82) observations for Δ*F* and 142 MUs with an average of 2.91 (SD: 1.74) observations for brace height, acceleration slope, attenuation slope, and angle. Across MUs, we did not find the coefficient of variation in measurements for Δ*F* and brace height to be significantly different from those seen with the torque at which a MU was recruited. This indicates that the inherent variability of the MU, or our ability to estimate its recruitment time, is no less than that of Δ*F* and brace height and implies that the variation in these values could be due to the variability of the MU. Of note, angle proved remarkably stable with a significantly lower coefficient of variation than all other metrics, including torque at MU recruitment. This is likely an artifact of its considerably higher average values.

### 4.4. Considerations/limitations

While we found brace height, acceleration slope, attenuation slope, and angle to detect changes in neuromodulatory and inhibitory drive to a simulated motor pool and provide reliable measures in human MUs, a few considerations and limitations must be noted. In particular, the inhibitory patterns investigated in the simulations are changes in the excitation–inhibition coupling (reciprocal, proportional). Such a dynamic change in inhibitory pattern to the motor pool may be highly speculative at this point, though recent work could point to potential mechanisms (Glover and Baker 2022). Regardless, one must consider that observed changes in Δ*F* could also be due to changes in excitation–inhibition coupling. Additionally, one may question the practicality of the range of neuromodulatory parameters employed in the simulations. As detailed, changes in neuromodulatory command were achieved by adjusting the maximum conductance of the L-type Ca channels that simulate intrinsic activation by PICs. Such an approach has been thoroughly characterized previously and will not be belabored here (Powers *et al* 2012, Powers and Heckman 2015, 2017). That said, the Δ*F* values observed in the simulated data do span the range of Δ*F* values commonly observed in experimental human studies (Oya *et al* 2009, Udina *et al* 2010, Kim *et al* 2020, Hassan *et al* 2021).

Insensitivity to inhibitory pattern may be a beneficial quality of brace height, as it could allow changes in inhibitory pattern and neuromodulatory drive to be isolated, but one does question how such a relation arises. Prior work with similarly simulated motoneurons has noted that changes in inhibitory profile, akin to this study, alter a motoneurons discharge rate at recruitment and peak, leading to changes in rate modulation and attenuation slope that may impact Δ*F* estimates (Powers *et al* 2012, Powers and Heckman 2017). Indeed, this is corroborated in our results that show the inhibitory profile to be a significant predictor of attenuation slope. In contrast to Δ*F*, the brace height we present is normalized to a theoretical maximum activation by PICs, accounting for changes in rate modulation and peak discharge rate. We speculate that this isolates the neuromodulatory effects of PICs and accounts for inhibitory driven changes, making brace height a better indicator of neuromodulation alone. Normalization of Δ*F* to account for changes in discharge rate at recruitment or peak may similarly isolate estimates and has been performed previously (Oya *et al* 2009).

Additional effort that expands upon the work presented here is certainly warranted. In specific, additional investigation utilizing biasing offsets of inhibition is warranted to both investigate the effects of tonic inhibitory input on the proposed metrics and alleviate the floor effect observed with Δ*F*. Additionally, the simulations presented here do not vary afterhyperpolarization duration or PIC voltage threshold, two factors that may explain observed differences in discharge rates between muscles and are unlikely to be quantified with brace height or Δ*F*. Furthermore, the proposed method of brace height quantification determines the maximum deviation of linearity in MU discharge from recruitment to peak discharge and thus may fail to adequately characterize the behavior of all MUs. Specifically, MUs that quickly achieve peak discharge shortly after recruitment and display a slow decrease in discharge until peak torque (i.e. decreasing discharge rate with increasing excitatory command) may not be thoroughly characterized. To overcome this limitation, a third region could be defined from peak discharge to peak torque and its slope quantified. This may provide additional information for units that more frequently display this phenomenon during triangular ramp contractions (e.g. shoulder muscles, SOL, etc.). Lastly, more rigorous exclusion criteria may be warranted to better isolate changes in neuromodulation or inhibitory profile and remove erroneous estimates.

## 5. Conclusions

In the present study, we employ a geometric approach for estimating neuromodulatory and inhibitory contributions to motoneuron discharge, by exploiting discharge non-linearities introduced by PICs. In specific, we detail methods for characterizing the ascending phase of MU discharge during time-varying linear tasks, including the deviation from linear discharge (i.e. brace height) and the rate of change in discharge (i.e. acceleration slope, attenuation slope, angle). We further characterize these metrics on a large human MU dataset and a simulated motoneuron pool with known excitatory, inhibitory, and neuromodulatory inputs. Using known inputs, we found brace height and attenuation slope to consistently represent neuromodulation and the pattern of inhibition to the motoneuron pool, respectively. In contrast, we found the paired MU analysis technique (Δ*F*) to depend on both neuromodulation and the pattern of inhibition supplied to the motoneuron pool. Spanning both the simulated motoneurons and human MUs, the quantification of brace height supplies an intuitive and computationally inexpensive method for achieving graded estimates of neuromodulatory and inhibitory drive to MUs on a single unit level. Using brace height and its associated metrics provides a complementary view to the commonly used paired analysis technique and generates a potential avenue for decoupling changes in neuromodulatory drive and the profile of inhibitory input (excitation–inhibition coupling).

## Data Availability

The data that support the findings of this study are available upon reasonable request from the authors.

## References

[jneacb1d7bib1] Afsharipour B, Manzur N, Duchcherer J, Fenrich K F, Thompson C K, Negro F, Quinlan K A, Bennett D J, Gorassini M A (2020). Estimation of self-sustained activity produced by persistent inward currents using firing rate profiles of multiple motor units in humans. J. Neurophysiol..

[jneacb1d7bib2] Bates D, Mächler M, Bolker B, Walker S (2015). Fitting linear mixed-effects models using lme4. J. Stat. Soft..

[jneacb1d7bib3] Beauchamp J A, Khurram O U, Dewald J, Heckman C J, Pearcey G (2022). A computational approach for generating continuous estimates of motor unit discharge rates and visualizing population discharge characteristics. J. Neural Eng..

[jneacb1d7bib4] Beauchamp J, Urday S, Plaisier T, Heckman C J, Dewald J (2022). Acute attenuation of motor impairments with a noradrenergic pharmacological agent in chronic hemiparetic stroke. Arch. Phys. Med. Rehabil..

[jneacb1d7bib5] Bennett D J, Li Y, Harvey P J, Gorassini M (2001). Evidence for plateau potentials in tail motoneurons of awake chronic spinal rats with spasticity. J. Neurophysiol..

[jneacb1d7bib6] Binder M D, Powers R K, Heckman C J (2020). Nonlinear input-output functions of motoneurons. Physiology.

[jneacb1d7bib7] Boccia G, Martinez-Valdes E, Negro F, Rainoldi A, Falla D (2019). Motor unit discharge rate and the estimated synaptic input to the vasti muscles is higher in open compared with closed kinetic chain exercise. J. Appl. Physiol..

[jneacb1d7bib8] Bowker R M, Westlund K N, Sullivan M C, Coulter J D (1982). Organization of descending serotonergic projections to the spinal cord. Prog. Brain Res..

[jneacb1d7bib9] Crone C, Hultborn H, Kiehn O, Mazieres L, Wigstrom H (1988). Maintained changes in motoneuronal excitability by short-lasting synaptic inputs in the decerebrate cat. J. Physiol..

[jneacb1d7bib10] De Luca C J, Contessa P (2012). Hierarchical control of motor units in voluntary contractions. J. Neurophysiol..

[jneacb1d7bib11] De Luca C J, Lefever R S, Mccue M P, Xenakis A P (1982). Behaviour of human motor units in different muscles during linearly varying contractions. J. Physiol..

[jneacb1d7bib12] Del Vecchio A, Casolo A, Negro F, Scorcelletti M, Bazzucchi I, Enoka R, Felici F, Farina D (2019). The increase in muscle force after 4 weeks of strength training is mediated by adaptations in motor unit recruitment and rate coding. J. Physiol..

[jneacb1d7bib13] Del Vecchio A, Holobar A, Falla D, Felici F, Enoka R, Farina D (2020). Tutorial: analysis of motor unit discharge characteristics from high-density surface EMG signals. J. Electromyogr. Kinesiol..

[jneacb1d7bib14] Elbasiouny S M, Bennett D J, Mushahwar V K (2006). Simulation of Ca_2+_ persistent inward currents in spinal motoneurones: mode of activation and integration of synaptic inputs. J. Physiol..

[jneacb1d7bib15] Elliott P, Wallis D I (1992). Serotonin and l-norepinephrine as mediators of altered excitability in neonatal rat motoneurons studied *in vitro*. Neuroscience.

[jneacb1d7bib16] Farina D, Holobar A, Gazzoni M, Zazula D, Merletti R, Enoka R M (2009). Adjustments differ among low-threshold motor units during intermittent, isometric contractions. J. Neurophysiol..

[jneacb1d7bib17] Fedirchuk B, Dai Y (2004). Monoamines increase the excitability of spinal neurones in the neonatal rat by hyperpolarizing the threshold for action potential production. J. Physiol..

[jneacb1d7bib18] Fuglevand A J, Lester R A, Johns R K (2015). Distinguishing intrinsic from extrinsic factors underlying firing rate saturation in human motor units. J. Neurophysiol..

[jneacb1d7bib19] Glover I S, Baker S N (2022). Both corticospinal and reticulospinal tracts control force of contraction. J. Neurosci..

[jneacb1d7bib20] Gorassini M A, Bennett D J, Yang J F (1998). Self-sustained firing of human motor units. Neurosci. Lett..

[jneacb1d7bib21] Gorassini M, Yang J F, Siu M, Bennett D J (2002). Intrinsic activation of human motoneurons: possible contribution to motor unit excitation. J. Neurophysiol..

[jneacb1d7bib22] Harvey P J, Li X, Li Y, Bennett D J (2006a). Endogenous monoamine receptor activation is essential for enabling persistent sodium currents and repetitive firing in rat spinal motoneurons. J. Neurophysiol..

[jneacb1d7bib23] Harvey P J, Li Y, Li X, Bennett D J (2006b). Persistent sodium currents and repetitive firing in motoneurons of the sacrocaudal spinal cord of adult rats. J. Neurophysiol..

[jneacb1d7bib24] Hassan A S, Fajardo M E, Cummings M, Mcpherson L M, Negro F, Dewald J P A, Heckman C J, Pearcey G E P (2021). Estimates of persistent inward currents are reduced in upper limb motor units of older adults. J. Physiol..

[jneacb1d7bib25] Hassan A, Thompson C K, Negro F, Cummings M, Powers R K, Heckman C J, Dewald J P A, Mcpherson L M (2020). Impact of parameter selection on estimates of motoneuron excitability using paired motor unit analysis. J. Neural. Eng..

[jneacb1d7bib26] Heckman C J, Enoka R M (2012). Motor unit. Compr. Physiol..

[jneacb1d7bib27] Heckman C J, Johnson M, Mottram C, Schuster J (2008). Persistent inward currents in spinal motoneurons and their influence on human motoneuron firing patterns. The Neuroscientist.

[jneacb1d7bib28] Hines M L, Carnevale N T (1997). The neuron simulation environment. Neural Comput..

[jneacb1d7bib29] Holstege J C, Kuypers H G J M (1987). Brainstem projections to spinal motoneurons: an update. Neuroscience.

[jneacb1d7bib30] Hounsgaard J, Hultborn H, Jespersen B, Kiehn O (1988). Bistability of alpha-motoneurones in the decerebrate cat and in the acute spinal cat after intravenous 5-Hydroxytryptophan. J. Physiol..

[jneacb1d7bib31] Hounsgaard J, Kiehn O (1985). Ca++ dependent bistability induced by serotonin in spinal motoneurons. Exp. Brain Res..

[jneacb1d7bib32] Hsiao C F, Trueblood P R, Levine M S, Chandler S H (1997). Multiple effects of serotonin on membrane properties of trigeminal motoneurons *in vitro*. J. Neurophysiol..

[jneacb1d7bib33] Hug F, Avrillon S, Del Vecchio A, Casolo A, Ibanez J, Nuccio S, Rossato J, Holobar A, Farina D (2021). Analysis of motor unit spike trains estimated from high-density surface electromyography is highly reliable across operators. J. Electromyogr. Kinesiol..

[jneacb1d7bib34] Johnson M D, Thompson C K, Tysseling V M, Powers R K, Heckman C J (2017). The potential for understanding the synaptic organization of human motor commands via the firing patterns of motoneurons. J. Neurophysiol..

[jneacb1d7bib35] Khurram O U, Pearcey G E P, Chardon M K, Kim E H, Garcia M, Heckman C J (2022). The cellular basis for the generation of firing patterns in human motor units. Adv. Neurobiol..

[jneacb1d7bib36] Kiehn O, Eken T (1997). Prolonged firing in motor units: evidence of plateau potentials in human motoneurons?. J. Neurophysiol..

[jneacb1d7bib37] Kim E H, Wilson J M, Thompson C K, Heckman C J (2020). Differences in estimated persistent inward currents between ankle flexors and extensors in humans. J. Neurophysiol..

[jneacb1d7bib38] Kim H, Major L A, Jones K E (2009). Derivation of cable parameters for a reduced model that retains asymmetric voltage attenuation of reconstructed spinal motor neuron dendrites. J. Comput. Neurosci..

[jneacb1d7bib39] Kirk E A, Zero A M, Rice C L (2021). Firing rate trajectories of human occipitofrontalis motor units in response to triangular voluntary contraction intensity. Exp. Brain Res..

[jneacb1d7bib40] Kuo J J, Lee R H, Zhang L, Heckman C J (2006). Essential role of the persistent sodium current in spike initiation during slowly rising inputs in mouse spinal neurones. J. Physiol..

[jneacb1d7bib41] Lee R H, Heckman C J (1996). Influence of voltage-sensitive dendritic conductances on bistable firing and effective synaptic current in cat spinal motoneurons *in vivo*. J. Neurophysiol..

[jneacb1d7bib42] Lee R H, Heckman C J (1998a). Bistability in spinal motoneurons *in vivo*: systematic variations in persistent inward currents. J. Neurophysiol..

[jneacb1d7bib43] Lee R H, Heckman C J (1998b). Bistability in spinal motoneurons *in vivo*: systematic variations in rhythmic firing patterns. J. Neurophysiol..

[jneacb1d7bib44] Lee R H, Heckman C J (1999a). Enhancement of bistability in spinal motoneurons *in vivo* by the noradrenergic Alpha1 agonist methoxamine. J. Neurophysiol..

[jneacb1d7bib45] Lee R H, Heckman C J (1999b). Paradoxical effect of Qx-314 on persistent inward currents and bistable behavior in spinal motoneurons *in vivo*. J. Neurophysiol..

[jneacb1d7bib46] Lee R H, Heckman C J (2000). Adjustable amplification of synaptic input in the dendrites of spinal motoneurons *in vivo*. J. Neurosci..

[jneacb1d7bib47] Lee R H, Heckman C J (2001). Essential role of a fast persistent inward current in action potential initiation and control of rhythmic firing. J. Neurophysiol..

[jneacb1d7bib48] Lenth Russell V (2022). emmeans: Estimated marginal means, aka least-squares means. R package version 1.7.5.

[jneacb1d7bib49] Li S, Chen Y-T, Francisco G E, Zhou P, Rymer W Z (2019). A unifying pathophysiological account for post-stroke spasticity and disordered motor control. Front. Neurol..

[jneacb1d7bib50] Li Y, Bennett D J (2003). Persistent sodium and calcium currents cause plateau potentials in motoneurons of chronic spinal rats. J. Neurophysiol..

[jneacb1d7bib51] Li Y, Gorassini M A, Bennett D J (2004). Role of persistent sodium and calcium currents in motoneuron firing and spasticity in chronic spinal rats. J. Neurophysiol..

[jneacb1d7bib52] Martinez-Valdes E, Negro F, Laine C M, Falla D, Mayer F, Farina D (2017). Tracking motor units longitudinally across experimental sessions with high-density surface electromyography. J. Physiol..

[jneacb1d7bib53] Mcauliffe D, Kmiec T, Taylor C, Thompson C (2020). Non-linear discharge of human motor units during linear time-varying contractions across motor pools.

[jneacb1d7bib54] Mcpherson J G, Ellis M D, Harden R N, Carmona C, Drogos J M, Heckman C J, Dewald J P A (2018). Neuromodulatory inputs to motoneurons contribute to the loss of independent joint control in chronic moderate to severe hemiparetic stroke. Front. Neurol..

[jneacb1d7bib55] Moritz A T, Newkirk G, Powers R K, Binder M D (2007). Facilitation of somatic calcium channels can evoke prolonged tail currents in rat hypoglossal motoneurons. J. Neurophysiol..

[jneacb1d7bib56] Murray K C (2010). Recovery of motoneuron and locomotor function after spinal cord injury depends on constitutive activity in 5-Ht2c receptors. Nat. Med..

[jneacb1d7bib57] Negro F, Muceli S, Castronovo A M, Holobar A, Farina D (2016). Multi-channel intramuscular and surface EMG decomposition by convolutive blind source separation. J. Neural. Eng..

[jneacb1d7bib58] Orssatto L B R, Mackay K, Shield A J, Sakugawa R L, Blazevich A J, Trajano G S (2021). Estimates of persistent inward currents increase with the level of voluntary drive in low-threshold motor units of plantar flexor muscles. J. Neurophysiol..

[jneacb1d7bib59] Oya T, Riek S, Cresswell A G (2009). Recruitment and rate coding organisation for soleus motor units across entire range of voluntary isometric plantar flexions. J. Physiol..

[jneacb1d7bib60] Pearcey G E P, Khurram O U, Beauchamp J A, Negro F, Heckman C J (2022). Antagonist tendon vibration dampens estimates of persistent inward currents in motor units of the human lower limb.

[jneacb1d7bib61] Perrier J-F, Hounsgaard J (2003). 5-Ht2 receptors promote plateau potentials in turtle spinal motoneurons by facilitating an L-type calcium current. J. Neurophysiol..

[jneacb1d7bib62] Person R S, Kudina L P (1972). Discharge frequency and discharge pattern of human motor units during voluntary contraction of muscle. Electroencephalogr. Clin. Neurophysiol..

[jneacb1d7bib63] Powers R K, Binder M D (2001). Input-output functions of mammalian motoneurons. Rev. Physiol. Biochem. Pharmacol..

[jneacb1d7bib64] Powers R K, Elbasiouny S M, Rymer W Z, Heckman C J (2012). Contribution of intrinsic properties and synaptic inputs to motoneuron discharge patterns: a simulation study. J. Neurophysiol..

[jneacb1d7bib65] Powers R K, Heckman C J (2015). Contribution of intrinsic motoneuron properties to discharge hysteresis and its estimation based on paired motor unit recordings: a simulation study. J. Neurophysiol..

[jneacb1d7bib66] Powers R K, Heckman C J (2017). Synaptic control of the shape of the motoneuron pool input-output function. J. Neurophysiol..

[jneacb1d7bib67] Powers R K, Nardelli P, Cope T C (2008). Estimation of the contribution of intrinsic currents to motoneuron firing based on paired motoneuron discharge records in the decerebrate cat. J. Neurophysiol..

[jneacb1d7bib68] R Core Team (2021). R: A language and environment for statistical computing. R Foundation for Statistical Computing.

[jneacb1d7bib69] Revill A L, Fuglevand A J (2017). Inhibition linearizes firing rate responses in human motor units: implications for the role of persistent inward currents. J. Physiol..

[jneacb1d7bib70] Sherrington C S (1907). On reciprocal innervation of antagonistic muscles.─tenth note. Proc. R. Soc. B.

[jneacb1d7bib71] Stephenson J L, Maluf K S (2011). Dependence of the paired motor unit analysis on motor unit discharge characteristics in the human tibialis anterior muscle. J. Neurosci. Methods.

[jneacb1d7bib72] Svirskis G, Hounsgaard J (1997). Depolarization-induced facilitation of a plateau-generating current in ventral horn neurons in the turtle spinal cord. J. Neurophysiol..

[jneacb1d7bib73] Udina E, D’amico J, Bergquist A J, Gorassini M A (2010). Amphetamine increases persistent inward currents in human motoneurons estimated from paired motor-unit activity. J. Neurophysiol..

[jneacb1d7bib74] Wada T, Hasegawa Y, Ono H (1997). Characterization of A1-adrenoceptor subtypes in facilitation of rat spinal motoneuron activity. Eur. J. Pharmacol..

[jneacb1d7bib75] Walton C, Kalmar J M, Cafarelli E (2002). Effect of caffeine on self-sustained firing in human motor units. J. Physiol..

[jneacb1d7bib76] Wilson J M, Thompson C K, Miller L C, Heckman C J (2015). Intrinsic excitability of human motoneurons in biceps brachii versus triceps brachii. J. Neurophysiol..

[jneacb1d7bib77] Zero A M, Kirk E A, Rice C L (2022). Firing rate trajectories of human motor units during activity-dependent muscle potentiation. J. Appl. Physiol..

